# Modelling trachoma post-2020: opportunities for mitigating the impact of COVID-19 and accelerating progress towards elimination

**DOI:** 10.1093/trstmh/traa171

**Published:** 2021-02-17

**Authors:** Anna Borlase, Seth Blumberg, E Kelly Callahan, Michael S Deiner, Scott D Nash, Travis C Porco, Anthony W Solomon, Thomas M Lietman, Joaquin M Prada, T Dèirdre Hollingsworth

**Affiliations:** Big Data Institute, Li Ka Shing Centre for Health Information and Discovery, University of Oxford, Oxford, UK; Francis I Proctor Foundation, UCSF, USA; Trachoma Control Program, The Carter Center, Atlanta, Georgia, USA; Francis I Proctor Foundation, UCSF, USA; Trachoma Control Program, The Carter Center, Atlanta, Georgia, USA; Francis I Proctor Foundation, UCSF, USA; Department of Control of Neglected Tropical Diseases, World Health Organisation, Geneva, Switzerland; Francis I Proctor Foundation, UCSF, USA; Faculty of Health and Medical Sciences, University of Surrey, UK; Big Data Institute, Li Ka Shing Centre for Health Information and Discovery, University of Oxford, Oxford, UK

**Keywords:** COVID-19, elimination, modelling, trachoma

## Abstract

**Background:**

The COVID-19 pandemic has disrupted planned annual antibiotic mass drug administration (MDA) activities that have formed the cornerstone of the largely successful global efforts to eliminate trachoma as a public health problem.

**Methods:**

Using a mathematical model we investigate the impact of interruption to MDA in trachoma-endemic settings. We evaluate potential measures to mitigate this impact and consider alternative strategies for accelerating progress in those areas where the trachoma elimination targets may not be achievable otherwise.

**Results:**

We demonstrate that for districts that were hyperendemic at baseline, or where the trachoma elimination thresholds have not already been achieved after three rounds of MDA, the interruption to planned MDA could lead to a delay to reaching elimination targets greater than the duration of interruption. We also show that an additional round of MDA in the year following MDA resumption could effectively mitigate this delay. For districts where the probability of elimination under annual MDA was already very low, we demonstrate that more intensive MDA schedules are needed to achieve agreed targets.

**Conclusion:**

Through appropriate use of additional MDA, the impact of COVID-19 in terms of delay to reaching trachoma elimination targets can be effectively mitigated. Additionally, more frequent MDA may accelerate progress towards 2030 goals.

## Introduction

In response to the COVID-19 pandemic, on 1 April 2020 the World Health Organization (WHO) released interim guidance that community-based surveys, active case-finding and mass drug administration (MDA) programmes for neglected tropical diseases (NTDs), including trachoma, be postponed.^1^

The active trachoma threshold for elimination as a public health problem (EPHP) has been set by WHO as a prevalence of trachomatous inflammation–follicular (TF) in children aged 1–9 y of less than 5% (TF_1–9_<5%).^2^ Annual district-level MDA of oral azithromycin, which targets the whole community, has formed the mainstay of the multifaceted global efforts to achieve this EPHP goal,^3^ with a single dose demonstrated to have good efficacy against ocular strains of *Chlamydia trachomatis*, the causative agent of trachoma.^4^ This strategy has proved to be widely successful in community-randomized studies^5,6^ and confirmed by the growing number of previously endemic countries, which are now reaching the active trachoma EPHP threshold.^3^

Given the pivotal role of annual MDA in the progress made in recent years, there are growing concerns regarding the potential impact of interruption to programmatic activities due to COVID-19.^7–9^ Empirical studies have indicated that upon cessation of MDA, infection returns exponentially in some areas, with a rate of resurgence anticipated to be faster in higher transmission settings.^10,11^ In the context of programmatic activities being temporarily halted due to COVID-19, this is especially concerning for districts with previously high prevalence of trachoma that are midway through elimination programmes. Furthermore, for a limited number of districts, the reproductive number under annual treatment (*R_T_*) has been estimated as being >1 (defined as ‘MDA super-critical’ by Blumberg et al.),^12^ suggesting that for this minority of districts, elimination is not achievable under a strategy of annual MDA.^12,13^ For such districts there is concern that the interruption to programmes may cause a particularly marked surge in transmission with unknown consequences for morbidity. Regardless of the impact of COVID-19, these districts may need to implement alternative control strategies if EPHP is to be achieved.^12^

Building on previously developed mathematical models for trachoma transmission,^14–16^ we explore the impact of an interruption to community-level MDA in a range of endemic settings. Our individual-based stochastic model incorporates some key aspects of ocular *C. trachomatis* infection biology, including acquired immunity leading to decreased duration of infection with repeated infection and allows simulation of some of the variability in response to MDA observed in empirical studies.^4,10,17,18^ We also consider the effect of additional rounds of MDA in the year following the resumption of activities as a potential mitigation strategy. For those settings where reaching the EPHP target may not be achievable under current practice, we then explore the potential effectiveness of enhanced MDA protocols beyond 2020, as possible strategies for both mitigation and acceleration towards EPHP targets.

## Methods

### Model structure

The model for *C. trachomatis* transmission is based on a previously described framework.^15,16^ The original population-based, deterministic model based on ordinary differential equations has subsequently been adapted to be stochastic^14^ and then further developed here to a fully stochastic individual-based model. The model incorporates current knowledge of the natural history and transmission of trachoma, including direct person-to-person transmission with infectivity proportional to an individual's bacterial load, children acting as a core group for transmission, individuals being susceptible to repeated infections and the persistence of TF after clearance of ocular *C. trachomatis* infection.^18–20^

**Figure 1.**
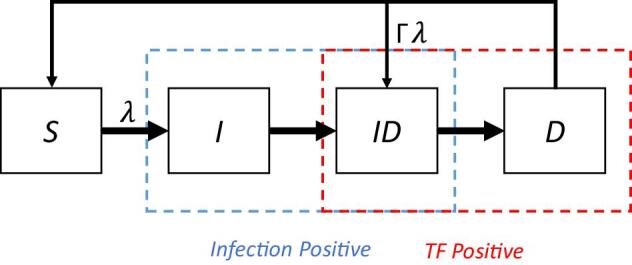
Schematic of model structure. Individuals can be susceptible to infection (S), infected but not yet diseased (I), infected and diseased (ID) or diseased but having cleared infection (D), where disease refers to trachomatous inflammation—follicular (TF). Individuals for whom infection has been cleared but disease persists (D) can be reinfected with force of infection ($\lambda )\ $reduced by $\Gamma $.

Individuals transition through four sequential states: susceptible (S), infected but not yet diseased (I), infected and diseased (ID) or diseased but no longer infected (D), as illustrated in Figure 1. Here, disease refers to active trachoma, specifically TF. Within this framework, people who have cleared infection but remain diseased (D) are susceptible to infection but with the force of infection ($\lambda $) reduced by a factor ($\Gamma $). The majority of model parameters are drawn from the literature, with the transmission parameter varied to represent different endemic settings. Further model description and model parameter definitions, values and sources are given in Supplementary Table S1.

Empirical studies have indicated that bacterial load, duration of infection and duration of clinical disease decrease with age and history of infection in trachoma-endemic communities.^4,18,20^ These aspects are represented within the model framework, with bacterial load, duration of ID and D for each individual assumed to decrease with each subsequent infection following negative exponentials.^14,16^ Age, current infection/disease status and total number of infections for each individual are explicitly incorporated; the model runs in 1-wk time steps.

### Treatment and systematic non-adherence

Community-wide MDA is assumed to be delivered to all ages with an 80% coverage level, in line with WHO minimum target coverage,^2^ assuming an efficacy (the probability that an individual who receives MDA clears infection) of 85%.^21^ To simulate the potentially lower efficacy of topical tetracycline eye ointment (which is routinely given to children aged <6 mo), treatment is assumed to be 50% less effective in this age group.

To account for the possible role of systematic non-adherence to MDA, the ‘controlled correlation’ method proposed by Dyson et al. is incorporated into the model (described in Supplementary Data).^22^ In simulations where *ρ* = 0, this is the equivalent to all rounds being randomly distributed (no systematic non-adherence, reflecting uncertainty regarding the presence of systematic non-adherence in trachoma control programmes);$\ \rho \ $ = 0.3 can be interpreted as a low level of systematic non-adherence and *ρ* = 0.5 is representative of an intermediate level of systematic non-adherence. Values above *ρ* = 0.5 (with the extreme value of *ρ* = 1 corresponding to complete systematic non-adherence, i.e. the same people being missed at every round) were not considered realistic for trachoma control programmes.

### Settings and simulated scenarios

**Figure 2.**
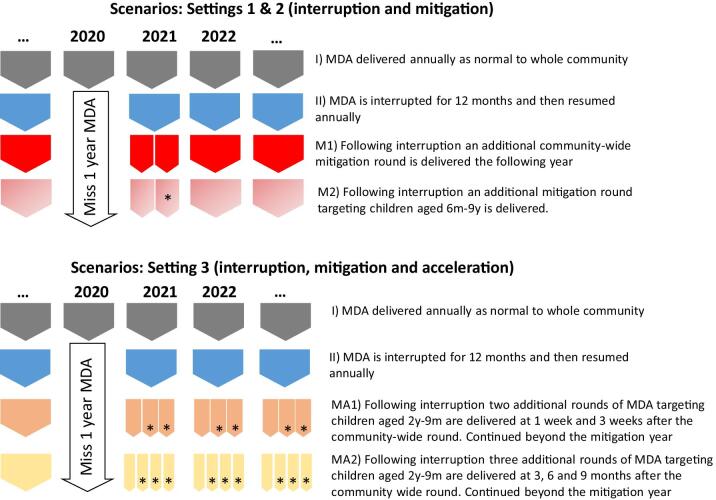
Simulated scenarios for Settings 1 and 2 (interruption and mitigation) and Setting 3 (interruption and mitigation/acceleration protocols). *denotes MDA round targeting children only.

Simulated scenarios are represented schematically in Figure 2.

#### Impact of 12-mo interruption

Trachoma-endemic settings are generally categorised as hypo-, meso- and hyperendemic, corresponding to TF prevalence in ages 1–9 y (TF_1–9_) of 5–9.9%, 10–29.9% and ≥30% respectively. We modelled the different settings by varying the transmission parameter, with simulations fitted to prevalence category at most recent survey. Here, we considered three alternatives: the most recent prevalence estimate could be either at baseline (i.e. before MDA has taken place) or at an impact survey after three or five rounds of annual MDA, reflecting WHO survey guidelines for trachoma programmes.^2^

The average impact of a 12-mo programme interruption (i.e. MDA is delivered 12 mo later than intended, equivalent to a 2-y gap between MDA rounds) was then estimated in terms of average delay to reaching the threshold of TF_1–9_<5%. The median delay is estimated as the difference between the median time to reaching the EPHP threshold after a 12-mo interruption and the median time to reaching the EPHP threshold if the interruption had not occurred (Supplementary Data Table S2).

Interruption was simulated to take place midway through a planned programme of MDA. This assumes a 3-y programme for mesoendemic settings (interruption in the second year), a 5-y programme for hyperendemic settings (interruption in the third year) and a 1-y programme for settings that were hypoendemic at the last survey (interruption in the first year following the most recent survey).^2,23^

#### Impact of mitigation and acceleration strategies

To evaluate in more detail the potential impact of implementing alternative MDA after a disruption to programmes, we consider three simulated settings with differing levels of transmission.

The first two settings (Setting 1 and Setting 2) were intended to represent districts that would have been expected to reach the TF_1–9_<5% threshold before 2030 with a strategy of annual district-level MDA targeting the whole community, but where an interruption is expected to cause a delay in achieving this control threshold. This corresponds to ‘MDA subcritical’ as defined by Blumberg et al.,^12^ with *R_0_*>1 and *R_T_*<1.

Setting 1 corresponds to a mean baseline (before MDA) TF_1–9_ of 40% (range 37.5–42.5) and Setting 2 corresponds to a mean baseline TF_1–9_ of 20% (range 17.5–22.5).

A third setting, denoted Setting 3, was simulated to represent those districts that after >10 y of annual MDA are still endemic (corresponding to ‘MDA supercritical’ as defined by Blumberg et al., with *R_T_*>1).^12^ This was done by simulating a higher level of transmission and also by filtering the stochastic simulations to include only those where TF_1–9_ is ≥10% after 10 annual rounds of MDA (see Supplementary Data for further details).

For Setting 1 and Setting 2, a 12-mo delay in MDA (yielding a 2-y gap in treatment) was simulated midway through a planned programme. We assumed a 5-y MDA programme for Setting 1 (interruption in year 3) and a 3-y MDA programme for Setting 2 (interruption in year 2).^2^ Two mitigation strategies that could be implemented after resuming activities were then considered:

**Mitigation protocol 1 (M1)**, an additional round of community-wide MDA (all ages) delivered 6 mo after the programme restarts;

**Mitigation protocol (M2)**, an additional round of MDA targeting only children 6mo-9y delivered 6 mo after the programme restarts.

For Setting 3, the 12-mo delay in MDA is simulated to take place in year 11. As this setting is intended to be representative of those settings in which previous models indicate a new paradigm may be needed to achieve EPHP, two alternative strategies are simulated. These are:

**Mitigation and acceleration protocol 1 (MA1)**, which consists of two extra rounds of MDA targeting children aged 2–9 y only, delivered 1 and 3 wk after the normal annual community-wide MDA; and

**Mitigation and acceleration protocol 2 (MA2)**, which consists of three extra rounds of MDA targeting children aged 2–9 y at 3-mo intervals following an annual community-wide MDA.

These are two strategies that have confirmed approval as clinical trial protocols in Ethiopia (clinicaltrials.gov identifier NCT03523156 for MA1; NCT03335072 for MA2).^24^

Given the current uncertainty regarding the global COVID-19 situation, further simulations representative of a 24-mo interruption to MDA (yielding a 3-y gap in treatment), followed by either no mitigation or mitigation strategies M1 and M2, were also carried out for Setting 1 and Setting 2.

A population of size 1000 was considered for all simulations, with average output from stochastic simulations considered representative of a district with a given level of transmission (further details in Supplementary Data). Simulations were run for 16 (Settings 1 and 2) or 23 y (Setting 3). Initial sets of stochastic simulations were filtered to give at least 1000 simulations for analysis based on criteria described (baseline prevalence or prevalence after a given number of MDA rounds). For Settings 1 and 2 (including mitigation simulations), to ensure simulations were representative of settings where TF_1–9_<5% would have been achievable by 2030, simulations which did not reach TF_1–9_<5% were also removed and not considered for analysis. The impact of systematic non-adherence was explored for Setting 3 by setting the adherence correlation parameter *ρ* to 0, 0.3 or 0.5. Confidence intervals are estimated as 95th centiles. All simulations were implemented in R version 3.6.3, with code available at https://github.com/AnnaMB123/AnnaMB123_TRSTMH_Trachoma.

### Results

**Figure 3.**
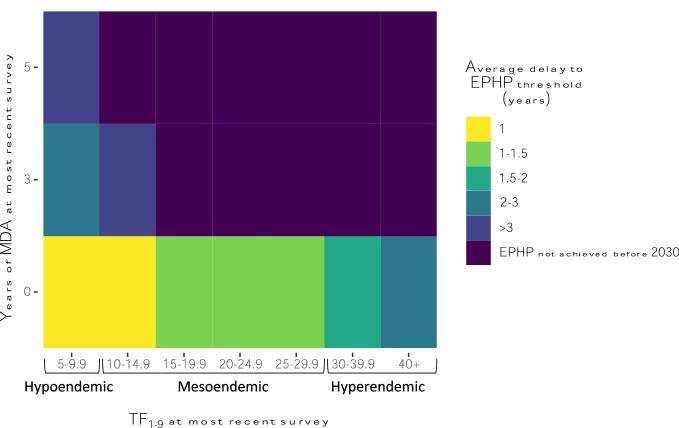
Median delay (years) to reaching EPHP threshold (TF_1–9_<5%) at varying levels of endemicity/stages of trachoma elimination programmes following a 1-y interruption to MDA. Years of MDA indicates the number of rounds of MDA that had been delivered when the last survey was carried out (0 representing baseline survey).

Figure 3 summarises the delay to programmes following a 1-y interruption across a range of endemic settings and levels of transmission, described by TF_1–9_ (x-axis) at the most recent survey, which may have been at baseline, after 3 y of MDA or after 5 y of MDA (y-axis; further results in Supplementary Data Table S2). As expected, higher baseline prevalence levels (which correspond to higher transmission settings) are more impacted by a 1-y interruption to MDA in terms of delay to reaching the EPHP threshold. In settings where the baseline prevalence was >40%, or where TF_1–9_ has not been achieved after 3 or 5 y of annual MDA, the delay to achieving the EPHP threshold is estimated to be substantially longer than the interruption. Furthermore, where TF_1–9_ is >15% after 3 y of MDA or >10% after 5 y of MDA, the median time to achieving the threshold is longer than the length of the simulation (16 y).

**Figure 4.**
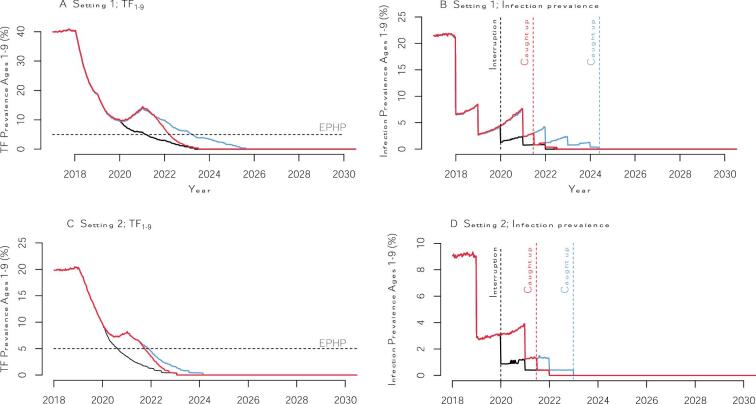
Median prevalence of TF in children aged 1–9 y (TF_1–9_; A and C) and ocular *Chlamydia trachomatis* infection (B and D). Setting 1 and Setting 2. The black curve represents no disruption to MDA (scenario I in Figure 2), the blue curve represents a 1-y interruption with no mitigation (scenario II in Figure 2) and the red curve represents an additional mitigation round of community-wide MDA given in the year following interruption (scenario M1 Figure 2).

Figure 4 (B and D) shows that with an additional round of community-wide MDA in the year following a 12-mo interruption (mitigation scenario M1), the prevalence of infection for both Setting 1 and Setting 2 is effectively reduced to where it would have been had the interruption not occurred. This has the effect of reducing the delay to achieving the EPHP threshold so that it is close to the duration of the interruption (12 mo) for both settings (Figure 4A,4B; Table 1). Similarly, after a 48-mo interruption (Supplementary Data Table S4), an additional community-based mitigation round in the year following programmes restarting will reduce the mean length of the delay to reaching the EPHP threshold to close to the 2-y duration of the interruption for both Settings 1 and 2. However, due to the slower bounce-back rate, the additional benefit of the mitigation round is not so pronounced in Setting 2.

**Table 1.** Summary of model output for Setting 1 and Setting 2: 12-mo interruption to MDA. Confidence intervals are given as 95th centiles

	Mean years to achieve EPHP (median; 95% CI)	% simulations reaching TF_1–9_<5% after 6 rounds of MDA	Mean % TF in children after 6 rounds MDA (median; 95% CI)
Setting 1			
(I) No interruption	4.3 (4.1; 2.4 to 11.1)	86.5	1.8 (0; 0 to 12.3)
(II) 2020 interruption; no mitigation	6.7 (6.2; 2.4 to >16^a^)	64.5	4.7 (0 to 20.9)
(M1) 2020 interruption; Extra MDA round: community	5.3 (5.3; 2.4 to >16^a^)	83.9	2.1 (0; 0 to 11.7)
(M2) 2020 interruption; extra MDA round: children	5.6 (5.4; 2.4 to >16^a^)	73.9	3.3 (1.3; 0 to 15.8)
Setting 2			
(I) No interruption	2.7 (2.6; 1.7 to 4.6)	98.6	0.8 (0.4; 0 to 4.2)
(II) 2020 interruption; no mitigation	4.1 (3.9; 1.7 to 6.2)	95.9	1.1 (0.4; 0 to 5.8)
(M1) 2020 interruption; extra MDA round: community	3.9 (3.8; 1.7 to 4.5)	98.9	0.9 (0.4; 0 to 3.8)
(M2) 2020 interruption; extra MDA round: children	3.9 (3.8; 1.7 to 5.3)	95.4	1.6 (1.2; 0 to 6.5)

Abbreviations: EPHP, elimination as a public health problem; MDA, mass drug administration.

^a^TF_1–9_ is not reached within the timescale of the simulations (16 y).

Following a 12-mo interruption, the impact of a mitigation round targeting children only (mitigation scenario M2; Table 1) in terms of delay and probability of reaching the threshold after a given number of MDA rounds is very similar to scenario M1, in which residents of all ages are offered antibiotics in the mitigation round. However, after a 2-y delay for Setting 1 (Supplementary Data Table S4), the mitigating effects of an extra round of MDA targeting only children are predicted to be less than if the whole community is targeted.

**Figure 5.**
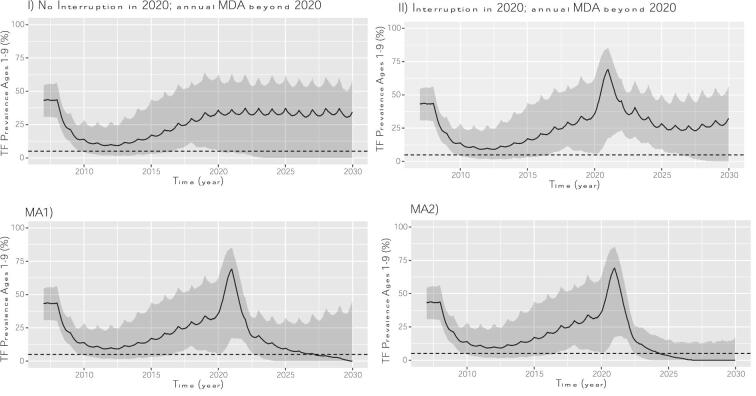
Median prevalence of TF in children aged 1–9 y (TF_1–9_) in Setting 3 (TF_1–9_>10% after 10 y of MDA). Adherence correlation parameter *ρ* = 0, corresponding to no systematic non-compliance. 95% confidence intervals are shown as shaded areas (estimated as 95th centiles). MA1 and MA2 represent mitigation and acceleration strategies described in Figure 2.

The simulations for Setting 3 (Figure 5 and Table 2) indicate that even without the interruption due to COVID-19 (scenario I), transmission levels in this setting are such that in the context of annual MDA, the probability of reaching the EPHP threshold for active trachoma is very low, even after 20 y of MDA and if no systematic non-adherence is assumed. In this setting, only 4.9% of simulations reach TF_1–9_<5% by 2030 when treatment is assumed to be random (adherence-correlation parameter *ρ* = 0) and only 0.9% if *ρ* = 0.5. Both the mitigation and acceleration strategies simulated (MA1 and MA2) show clear improvement in the probability of reaching the EPHP goal by 2030. Where no systematic non-adherence is assumed (*ρ* = 0), the median year by which TF_1–9_<5% would be reached is estimated to be 2027 for MA1 (representing 21 rounds of MDA post-2020) and 2024 for MA2 (after 14 rounds of MDA).

**Table 2.** Summary of model output for Setting 3 (TF_1–9_>10% after 10 y of MDA). Confidence intervals are given as 95th centiles

Setting 3	Adherence correlation parameter^a^$( \rho )$	% simulations reaching TF_1–9_<5% by 2030	Mean % TF_1–9_ in 2030 (median; 95% CI)
(I) No interruption	0	4.9	33.6 (34.5; 0 to 57.7)
	0.3	1.9	36.4 (36.9; 10.6 to 59.0)
	0.5	0.9	35.2 (35.2; 14.4 to 56.8)
(II) 2020 interruption; no mitigation, continue annual MDA beyond 2020	0	5.6	31.2 (32.4; 0 to 56.3)
	0.3	2.6	33.8 (34.2; 4.5 to 55.3)
	0.5	1.3	33.4 (33.6; 10.5 to 55.)
(MA1) 2020 interruption; mitigation and acceleration strategy 1	0	57.4	10.0 (0; 0 to 44.4)
	0.3	32.9	17.1 (15.9; 0 to 48.7)
	0.5	12.9	22.6 (22.7; 0 to 49.6)
(MA2) 2020 interruption; mitigation and acceleration strategy 2	0	90.3	1.6 (0; 0 to 16.7)
	0.3	67.9	4.7 (0; 0 to 24.5)
	0.5	38.9	8.9 (7.4; 0 to 28.0)

^a^The adherence correlation parameter $\rho $ is varied here to account for the possible role of systematic non-adherence to MDA. If $\rho $ = 0, this is the equivalent to all rounds being randomly distributed (no systematic non-adherence); $\rho $ = 0.3 can be interpreted as a low level of systematic non-adherence and $\rho $ = 0.5 can be interpreted as an intermediate level of systematic non-adherence. If $\rho $ = 1, this would correspond to the same individuals being missed at each round of MDA (complete systematic non-adherence).

These estimates are based on the assumption that these protocols are implemented in 2021 with the same minimum coverage level as before interruption, and continued until the TF_1–9_<5% threshold is achieved. For the mitigation and acceleration strategies MA1 and MA2, if systematic non-adherence is assumed to increase, the probability of reaching the threshold decreases, but remains markedly higher than that for continued annual MDA (Table 2).

## Discussion

Our results provide quantitative insights into the impact the current disruption to MDA activities may have on global efforts to eliminate trachoma, highlighting the need to prioritise mitigation strategies in those areas where the impact is predicted to be greatest. We also demonstrate the imperative need for alternative approaches in those areas where EPHP is unlikely to be achieved under existing protocols, notwithstanding the impact of COVID-19.

For those settings where EPHP would on average be achievable before 2030 (corresponding to *R_T_*<1 described by Blumberg et al.),^12^ but which were either hyperendemic at baseline (Setting 1) or where control has not been achieved after 3 y of MDA, our results indicate that missing a single round of treatment will lead to an increase in the number of MDA rounds needed to reach elimination targets. Following a 1-y interruption in these settings, it is estimated it will take on average >2 y to catch up if no mitigation protocols are implemented. By comparison, in Setting 2, where transmission is lower and baseline prevalence is mesoendemic (mean 20%), EPHP targets would still on average be reached after the same number of treatment rounds, and the length of the delay will be closer to the 1-y length of the simulated interruption. It is clear that as the level of transmission increases, the rate of resurgence during a period of programmatic interruption will also increase, which corresponds to the greater delay and number of treatment rounds necessary to make up lost ground once treatment is restarted. This is consistent with the expectation, supported by empirical evidence, that trachoma resurges more rapidly in higher prevalence settings.^10,11^

We also demonstrate that for both Setting 1 and Setting 2, a single additional round of MDA in the year following a 12-mo disruption will be sufficient to bring infection levels back to the level they would have been if the interruption had not occurred. The impact of this additional round is shown to be similar regardless of whether only children or the whole community are targeted. This is representative of the fact that in the model framework, in accordance with empirical evidence, children effectively act as a core group, due to both the higher bacterial loads and longer duration of infection that is assumed for early infections.^18,20^,^25–27^

It is noteworthy that for both Settings 1 and 2, even although mitigation MDA rounds bring infection levels back to where they would have been without interruption, this still leads to delay in achieving the EPHP threshold of approximately the same length as the interruption time period (just over 1 y). This is because the model represents the persistence of TF following resolution of infection. TF is a lagging indicator of infection at both the individual and population level, with correlation between TF and infection prevalence generally decreasing following MDA.^28^

With risk of donor fatigue a growing concern for many NTD control programmes,^29^ avoiding a prolonged delay to reaching EPHP targets is clearly a priority. Moreover, a delay to reaching trachoma thresholds will have a negative effect in terms of morbidity. Any surge in transmission during interruption, and every year of delay to achieving EPHP, corresponds to more *C. trachomatis* infections at both the individual and community level, the cumulative impact of which is more severe pathology and sequelae.^30^ While many trachoma programmes are making plans to restart MDA in 2021 with precautionary measures following updated guidance from WHO,^31^ if interruptions were to be even longer than 1 y, the predicted impact in terms of delays to achieving targets and longer term consequences for morbidity would be even greater.

In the mitigation scenarios explored here, additional catch-up rounds were simulated at 6 mo after the programme restarted; however, previous work suggests that regardless of timing, the anticipated mitigating effect of additional rounds of MDA delivered in the year following interruption would be equivalent.^32^ It is acknowledged that economical and operational constraints will play a key role in decision-making regarding implementation of any mitigation measures, with quantification of the economic costs attributable to programme interruptions in each trachoma-endemic region beyond the scope of this study. However, our results indicate that to minimise the impact both on morbidity and delay to achieving EPHP, additional rounds of MDA should be prioritised in high transmission settings, and delivered as soon as is practicable once programmes are able to restart at the same coverage levels as previously implemented.

While a single additional round of MDA may be sufficient in some settings to bring infection levels back to where they would have been without interruption, for those settings where current schedules would not achieve EPHP even if interruption had not occurred, this is clearly far from adequate.

The mitigation and acceleration strategies simulated here (MA1 and MA2) represent two potential new paradigms of treatment for these settings, with both predicting clear improvement when compared with annual MDA. Both strategies have confirmed approval as protocols for clinical trials in Ethiopia, where some districts may be similar to Setting 3. While model predictions for MA2 indicate an increased probability of achieving EPHP compared with MA1, and trials have already demonstrated the efficacy of a similar protocol,^5^ logistical and financial constraints mean quarterly treatment (MA2) may be less feasible than three closely spaced treatment rounds (MA1). Furthermore, uncertainty regarding the assumptions of the model, in particular regarding non-adherence, limits direct comparisons between the two protocols.

For both mitigation and acceleration strategies, if even a relatively low level of systematic non-compliance is assumed (*ρ* = 0.3), the probability of achieving EPHP is decreased, although it would still be considerably greater than with annual MDA (at least a 10-fold increase in probability of achieving EPHP for either MA1 or MA2). However, the model assumes that individuals who are unlikely to adhere to treatment recommendations are randomly distributed throughout the population,^22^ whereas in reality, it is not only how many people are repeatedly missed by MDA, but who are missed, that determines the overall success of a programme. A study of trachoma-endemic communities in Niger, for example, indicated that people who do not present for treatment are in fact less likely to be infected with ocular *C. trachomatis.*^33^ A study in Ethiopia indicated that levels of refusal were extremely low, at 0.6% of those offered azithromycin,^34^ with travelling during the campaign given as a major reason for non-treatment. Whether a protocol of repeated treatment rounds within a short space of time (MA1) or evenly spaced throughout the year (MA2) will maximise the probability of reaching all individuals targeted will likely be context-specific, but if systematically missed people are in fact less likely to be infected, the impact of non-adherence will be overestimated by the model. Another potentially important assumption of the model is that at the individual level, the infection-clearance outcome of receiving antibiotics is essentially binary and applied instantaneously. In reality, however, even if an individual does not clear infection after treatment, their bacterial load is likely to be reduced. The implication of this is that the model predictions for MA1, in which three MDA rounds are delivered very close to each other, are likely to be somewhat pessimistic. Further empirical data on heterogeneity of bacterial load and efficacy of treatment for a given bacterial load would improve the biological realism of this aspect of the model.

Within the model framework used here, the higher transmission rates and baseline prevalence levels are simulated by increasing the transmission parameter. This parameter does not have a directly interpretable meaning in itself but can be considered a proxy for the range of factors that facilitate transmission of ocular *C. trachomatis* infection. These include overcrowding, lack of access to clean water and comorbidities. The model does not currently incorporate the potential reduction in transmission afforded by facial cleanliness and environmental improvement interventions, which also form part of the WHO strategy for trachoma control in addition to MDA, due to uncertainty regarding their relative importance in reducing transmission.^2^ As such, the model predictions could be considered conservative. However, there are many potential indirect effects for COVID-19 on ocular *C. trachomatis* transmission in addition to the interruption to MDA that are as yet unknown. Given directives on physical distancing and the increased promotion of hygiene practices such as handwashing, it may be that transmission of *C. trachomatis* will decrease following COVID-19 in some settings. However, other possible consequences of COVID-19, such as migration, changes to health-seeking behaviour and economic hardship, may exacerbate the increases in infection predicted by the model.

### Conclusion

The COVID-19 pandemic represents an unprecedented challenge to communities and healthcare systems worldwide. While the current interruption to NTD control activities is clearly in line with the urgent need to minimise the spread of severe acute respiratory syndrome coronavirus 2 (SARS-CoV-2), it is crucial that trachoma programmes are resumed at the same coverage levels as before interruption, as soon as is practicable. Further work, for example, appraisal of previous survey data and estimation of *R_T_*, is needed to confirm which districts should be prioritised for additional mitigation rounds of MDA once programmes are able to resume, and also to identify those districts where mitigation and acceleration are needed. Despite the many challenges presented by COVID-19, the current interruption could potentially represent an opportunity to critically review trachoma programmes and revise protocols as we look towards 2030.

## Supplementary Material

traa171_Supplemental_File

## Data Availability

This work does not include analysis of new data; all code is available at https://github.com/AnnaMB123/AnnaMB123_TRSTMH_Trachoma.

## References

[1] World Health Organisation. COVID-19 Interim Guidance. Available at https://www.who.int/neglected_diseases/news/COVID19-WHO-interim-guidance-implementation-NTD-programmes/en/ [accessed August 6, 2020].

[2] World Health Organization. Report of the 3rd Global Scientific Meeting on Trachoma; 2010. Balitmore, USA: WHO. Available at https://www.who.int/blindness/publications/WORLDHEALTHORGANIZATIONGSMmtgreportFINALVERSION.pdf?ua=1 [accessed October 30, 2020].

[3] Lietman TM , Oldenburg CE, Keenan JD. Trachoma: Time to Talk Eradication. Ophthalmology. 2020;127(1):11–3. 31864470 10.1016/j.ophtha.2019.11.001

[4] Bailey RL , Arullendran P, Whittle HC et al. Randomised controlled trial of single-dose azithromycin in treatment of trachoma. Lancet. 1993;342(8869):453–6. 8102427 10.1016/0140-6736(93)91591-9

[5] House JI , Ayele B, Porco TC et al. Assessment of herd protection against trachoma due to repeated mass antibiotic distributions: a cluster-randomised trial. Lancet. 2009;373(9669):1111–8. 19329003 10.1016/S0140-6736(09)60323-8

[6] Chidambaram JD , Alemayehu W, Melese M et al. Effect of a single mass antibiotic distribution on the prevalence of infectious trachoma. JAMA. 2006;295(10):1142–6. 16522834 10.1001/jama.295.10.1142

[7] Ehrenberg JP , Zhou X-N, Fontes G et al. Strategies supporting the prevention and control of neglected tropical diseases during and beyond the COVID-19 pandemic. Infect Dis Poverty. 2020;9(1):86. 32646512 10.1186/s40249-020-00701-7 PMC7347419

[8] Molyneux DH , Aboe A, Isiyaku S et al. COVID-19 and neglected tropical diseases in Africa: impacts, interactions, consequences. Int Health. 2020;12(5):367–72. 32725145 10.1093/inthealth/ihaa040 PMC7443717

[9] Abdela SG , van Griensven J, Seife F et al. Neglecting the effect of COVID-19 on neglected tropical diseases: the Ethiopian perspective. Trans R Soc Trop Med Hyg. 2020;114(10):730–2. 32853370 10.1093/trstmh/traa072 PMC7499774

[10] Lakew T , House J, Hong KC et al. Reduction and return of infectious trachoma in severely affected communities in Ethiopia. PLoS Negl Trop Dis. 2009;3(2):e376. 19190781 10.1371/journal.pntd.0000376 PMC2632737

[11] Amza A , Kadri B, Nassirou B et al. Effectiveness of expanding annual mass azithromycin distribution treatment coverage for trachoma in Niger: a cluster randomised trial. Br J Ophthalmol. 2018;102(5):680–6. 28893761 10.1136/bjophthalmol-2017-310916 PMC5845832

[12] Blumberg S , Borlase A, Prada JM et al. Implications of the COVID-19 pandemic on eliminating trachoma as a public health problem. Under Rev. Published online 2020. 10.1093/trstmh/traa170 PMC7928550 33449114

[13] Lietman TM , Pinsent A, Liu F et al. Models of trachoma transmission and their policy implications: from control to elimination. Clin Infect Dis. 2018;66(suppl_4):S275–80. 29860288 10.1093/cid/ciy004 PMC5982784

[14] Godwin W , Prada JM, Emerson P et al. Trachoma prevalence after discontinuation of mass azithromycin distribution. J Infect Dis. 2020; 221(Supplement_5):S519–24. 32052842 10.1093/infdis/jiz691 PMC7289551

[15] Pinsent A , Gambhir M. Improving our forecasts for trachoma elimination: What else do we need to know? PLoS Negl Trop Dis. 2017;11(2):e0005378. 28182664 10.1371/journal.pntd.0005378 PMC5321453

[16] Pinsent A , Hollingsworth TD. Optimising sampling regimes and data collection to inform surveillance for trachoma control. PLoS Negl Trop Dis. 2018;12(10):e0006531. 30307939 10.1371/journal.pntd.0006531 PMC6181273

[17] Oldenburg CE , Amza A, Kadri B et al. Comparison of mass azithromycin coverage targets of children in Niger: A cluster-randomized trachoma trial. Am J Trop Med Hyg. 2018;98(2):389–95. 29260659 10.4269/ajtmh.17-0501 PMC5929194

[18] Bailey R , Duong T, Carpenter R et al. The duration of human ocular Chlamydia trachomatis infection is age dependent. Epidemiol Infect. 1999;123(3):479–86. 10694161 10.1017/s0950268899003076 PMC2810784

[19] Grassly NC , Ward ME, Ferris S et al. The natural history of trachoma infection and disease in a Gambian cohort with frequent follow-up. PLoS Negl Trop Dis. 2008;2(12):e341. 19048024 10.1371/journal.pntd.0000341 PMC2584235

[20] Solomon AW , Holland MJ, Burton MJ et al. Strategies for control of trachoma: observational study with quantitative PCR. Lancet. 2003;362(9379):198–204. 12885481 10.1016/S0140-6736(03)13909-8

[21] Liu F , Porco TC, Mkocha HA et al. The efficacy of oral azithromycin in clearing ocular chlamydia: mathematical modeling from a community-randomized trachoma trial. Epidemics. 2014;6:10–7. 24593917 10.1016/j.epidem.2013.12.001 PMC4420489

[22] Dyson L , Stolk WA, Farrell SH et al. Measuring and modelling the effects of systematic non-adherence to mass drug administration. Epidemics. 2017;18:56–66. 28279457 10.1016/j.epidem.2017.02.002 PMC5340860

[23] World Health Organization Technical advisory group on neglected tropical diseases. Technical Consultation on Trachoma Surveillance; 2014. Available at https://apps.who.int/iris/bitstream/handle/10665/174085/WHO_HTM_NTD_2015.02_en.pdf2015?sequence=1 [accessed October 30, 2020].

[24] Database of privately and publicly funded clinical studies around the world. Available at clinicaltrials.gov [accessed August 5, 2020].

[25] Nash SD , Chernet A, Moncada J et al. Ocular Chlamydia trachomatis infection and infectious load among pre-school aged children within trachoma hyperendemic districts receiving the SAFE strategy, Amhara region, Ethiopia. PLoS Negl Trop Dis. 2020;14(5):e0008226. 32421719 10.1371/journal.pntd.0008226 PMC7259799

[26] West ES , Munoz B, Mkocha H et al. Mass treatment and the effect on the load of Chlamydia trachomatis infection in a trachoma-hyperendemic community. Invest Ophthalmol Vis Sci. 2005;46(1):83–7. 15623758 10.1167/iovs.04-0327 PMC6853789

[27] Last A , Burr S, Alexander N et al. Spatial clustering of high load ocular Chlamydia trachomatis infection in trachoma: a cross-sectional population-based study. Pathog Dis. 2017;75(5):ftx050. 10.1093/femspd/ftx050 PMC5808645 28472466

[28] Ramadhani AM , Derrick T, Macleod D et al. The relationship between active trachoma and ocular Chlamydia trachomatis infection before and after mass antibiotic treatment. PLoS Negl Trop Dis. 2016;10(10):e0005080. 27783678 10.1371/journal.pntd.0005080 PMC5082620

[29] World Health Organization. End the neglect to attain the sustainable development goals - A Road Map for Neglected Tropical Diseases 2021–2030. Geneva: World Health Organization; 2020.

[30] Taylor HR , Burton MJ, Haddad D et al. Trachoma. Lancet. 2014;384(9960):2142–52. 25043452 10.1016/S0140-6736(13)62182-0

[31] World Health Organization. Considerations for Implementing Mass Treatment, Active Case-Finding and Population-Based Surveys for Neglected Tropical Diseases in The Context of the COVID-19 Pandemic. Geneva: World Health Organization; 2020.

[32] Gao D , Lietman TM, Dong C-P et al. Mass drug administration: the importance of synchrony. Math Med Biol. 2017;34(2):241–60. 27118395 10.1093/imammb/dqw005 PMC6201266

[33] Amza A , Kadri B, Nassirou B et al. The easiest children to reach are most likely to be infected with ocular Chlamydia trachomatis in trachoma endemic areas of Niger. PLoS Negl Trop Dis. 2013;7(1):e1983. 23326612 10.1371/journal.pntd.0001983 PMC3542188

[34] Astale T , Sata E, Zerihun M et al. Population-based coverage survey results following the mass drug administration of azithromycin for the treatment of trachoma in Amhara, Ethiopia. PLoS Negl Trop Dis. 2018;12(2):e0006270. 29451881 10.1371/journal.pntd.0006270 PMC5833287

