# Supplementary material for: Modelling trachoma post-2020: opportunities for mitigating the impact of COVID-19 and accelerating progress towards elimination
Source: Trans R Soc Trop Med Hyg. 2021 Feb 17;115(3):213–21. doi: 10.1093/trstmh/traa171 (PMC7928577; doi:10.1093/trstmh/traa171)
Supplement: traa171_Supplemental_File [file traa171_supplemental_file.docx]

**Supplementary Data.**

**Further details on transmission model.**

The model is based on a previously described framework^1^ which was validated and identified as the most parsimonious and best fit to cross-sectional PCR and TF data in a study comparing several possible frameworks for *C. trachomatis* transmission.^2^

The individual-based stochastic model described here incorporates some key aspects of ocular *C. trachomatis* infection biology, including acquired immunity leading to decreased duration of infection with repeated infection^3,4^ and allows simulation of some of the variability in response to MDA observed in empirical studies.^5,6^ Based on a modified SEIR framework (S=Susceptible; E=Exposed; I=Infectious; R=Recovered), individuals transition through four sequential states: Susceptible (S), infected but not yet diseased (I), infected and diseased (ID) or diseased but no longer infected (D). (See Figure 1 main text).

For each individual *i*, the duration of first ID and D periods (*ID_i,1_; D_i,1_*) are randomly assigned from Poisson distributions, with distribution means given as the baseline (longest) duration used by Pinsent and colleagues (see Table S1).^1^ The duration of these periods for subsequent infections are then assumed to decrease following a negative exponential to a minimum value, with decay rates and minimum durations also as given by Pinsent and colleagues.^1^ Similarly, it is assumed that an individual’s infectivity is proportional to their bacterial load, and that this also declines from the first infection following a negative exponential with each subsequent infection. For each individual’s (*i*) infection number (*j*), the calculated durations of *ID_i,j_* and *D_i,j_* are used as fixed transition periods, in contrast to exponential transitions utilised in the previous models.^1,7^ In order to ensure that the age-distribution of historical infections (and therefore infectivity, duration of infection and disease) are representative for a given level of transmission, a 40 year burn-in period was implemented for all simulations (burn-in period not considered for analyses).

A population size of 1000 was chosen, representing as small community (e.g. village). Preliminary simulations found this to provide equivalent results to a population of 8,000-10,000 individuals which is the smallest population unit (village) considered for the purposes of trachoma monitoring and evaluation.^8^ The relative role of different aspects of larger population structures (for example, worker networks) in trachoma transmission are not well quantified and likely to be setting-specific. However it is widely acknowledged that transmission of the ocular strains of *Chlamydia trachomatis* (the causative agent of trachoma) is predominantly via close contact and that children form the core group for transmission of trachoma.^9,10^ Therefore it was considered that the results from the population sizes simulated here, with age-disassortative mixing also incorporated into the model, would be broadly generalisable. In addition, the stochastic nature of the model (by simulating some of the variability in response to MDA observed in empirical studies^5,6^) means that averages of the model simulation outputs are considered to be as representative of a district with a given level of transmission as is practicable without incorporating specific metapopulation data.

To account for the possible role of systematic non-adherence to MDA, the “controlled correlation” method proposed by Dyson and colleagues is incorporated into the model.^11^ In this scheme, the first round of MDA is distributed randomly with probability given as the treatment coverage *c.* In each subsequent round *k*, individual *i* then receives treatment with probability *p_i,k_* given as:

$$p_{i,k}=(c\left( 1-\rho\right)+\rho R_{i})/(1+\left( k-2 \right)\rho)$$

Where *R_i_* is the total number of rounds attended by an individual previously, and $\rho$ is the correlation parameter. If $\rho$ =0, this is the equivalent to all rounds being randomly distributed (no systematic non-adherence) and if $\rho$ =1 this corresponds to only those individuals who received the first round of treatment receiving future rounds (complete systematic non-adherence).

Test uncertainty and decision-making around reaching the TF_1-9_ threshold (which would normally determine whether MDA is halted) was not considered here.

**Table S1. Model variables, parameters and sources.**

| **Notation** | **Description** | **Values /distribution** | **Units** | **Source** |
| --- | --- | --- | --- | --- |
| *S_i_* | Susceptible individuals | - |  |  |
| *I_i,j_* | Infected and not yet diseased  (individual *i* infection *j*) | 14 | Days | ^12^ |
| *ID_i,1_* | Infected and diseased period  (individual *i,* first infection) | $\sim Poisson(\omega_{max})$ | Days |  |
| *ID_i,j_* | Infected and diseased period  (individual *i,* infection *j*) | ${(ID}_{i,1}-\omega_{min} )e^{\phi(j-1)}+\omega_{min}$ | Days | ^1,2,12^ |
| *D_i,1_* | Diseased only period  (individual *i,* infection *j*) | $\sim Poisson(\tau_{max})$ | Days |  |
| *D_i,j_* | Diseased only period  (individual *i,* infection *j*) | ${(D}_{i,1}-\tau_{min} )e^{\theta(j-1)}+\tau_{min}$ | Days | ^1,2,12^ |
| $\omega$ | Duration of infected and diseased period | $\omega_{max}=200{, \omega}_{min}$=77 | Days | ^1,2,12^ |
| $\tau$ | Duration of diseased only period | $\tau_{max}=300{, \tau}_{min}$=7 | Days | ^1,2,12^ |
| $\phi$ | Decay rate, infected and diseased period | 0.45 | Proportion | ^1,2,12^ |
| $\theta$ | Decay rate, diseased only period | 0.3 | Proportion | ^1,2,12^ |
| *b* | Infectivity of an individual proportional to their bacterial load | 0.114 | Proportion | ^1,2^ |
| *β* | Transmission parameter | Varied to simulate a range of settings |  |  |
| $\lambda_{a}$ | Force of infection for age group *a* | Calculated | Weeks | ^1,2^ |
| $\Gamma$ | Reduction in force of infection during disease only state | 0.5 | Proportion | ^1,2,13^ |
| $c$ | Treatment coverage (proportion of population receiving treatment at each round of MDA) | 0.8 | Proportion | ^8^ |
| $\epsilon$ | Treatment efficacy (probability of infection clearance given that treatment is received) | 0.85 | Proportion | ^14^ |
| $\rho$ | Compliance correlation parameter | 0, 0.3 and 0.5 considered |  | ^11^ |

**Additional results: Estimating impact of 12 month interruption to MDA.**

Estimates are based on 1000 simulations for each category, filtered and sampled from an initial set of simulations to give a uniform distribution between category bounds.

Where the median/mean time to reaching the EPHP threshold without interruption to MDA is longer than the duration of the simulation (16 years), the delay cannot be estimated here and is given as NA.

**Table S2. Summary of the impact of a 12 month interruption to MDA on reaching EPHP threshold of TF_1-9_<5% at varying levels of endemicity/stages of trachoma elimination programmes.**

| **Years of MDA, most recent survey** | **TF_1-9,_**  **most recent**  **survey**  **%** | **Median time to EPHP threshold;**  **No interruption to MDA** | **Mean time to EPHP threshold;**  **No interruption to MDA** | **Median time to EPHP threshold;**  **12 month interruption to MDA** | **Mean time to EPHP threshold;**  **12 month interruption to MDA** | **Median**  **Delay to EPHP threshold** | **Mean**  **Delay to EPHP threshold** |
| --- | --- | --- | --- | --- | --- | --- | --- |
| 0 | 5-9.9 | 1.98 | 2.04 | 2.96 | 3 | 0.98 | 0.96 |
|  | 10-14.9 | 2.15 | 2.19 | 3.17 | 3.21 | 1.02 | 1.02 |
|  | 15-19.9 | 2.39 | 2.44 | 3.48 | 3.62 | 1.09 | 1.18 |
|  | 20-24.9 | 2.77 | 2.89 | 4.06 | 4.19 | 1.29 | 1.3 |
|  | 25-29.9 | 3.06 | 3.23 | 4.37 | 4.52 | 1.31 | 1.29 |
|  | 30-39.9 | 3.62 | 4.1 | 5.33 | 6.31 | 1.71 | 2.21 |
|  | 40+ | 4.6 | 5.31 | 7.15 | >16 | 2.55 | >9.69 |
| 3 | 5-9.9 | 4.6 | 4.77 | 7.06 | >16 | 2.46 | >10.23 |
|  | 10-14.9 | 6.75 | >16 | >16 | >16 | >8.25 | NA |
|  | 15-19.9 | >16 | >16 | >16 | >16 | NA | NA |
|  | 20-24.9 | >16 | >16 | >16 | >16 | NA | NA |
|  | 25-29.9 | >16 | >16 | >16 | >16 | NA | NA |
|  | 30-39.9 | >16 | >16 | >16 | >16 | NA | NA |
|  | 40+ | >16 | >16 | >16 | >16 | NA | NA |
| 5 | 5-9.9 | 7.31 | >16 | >16 | >16 | >7.69 | NA |
|  | 10-14.9 | >16 | >16 | >16 | >16 | NA | NA |
|  | 15-19.9 | >16 | >16 | >16 | >16 | NA | NA |
|  | 20-24.9 | >16 | >16 | >16 | >16 | NA | NA |
|  | 25-29.9 | >16 | >16 | >16 | >16 | NA | NA |
|  | 30-39.9 | >16 | >16 | >16 | >16 | NA | NA |
|  | 40+ | >16 | >16 | >16 | >16 | NA | NA |

**Additional results: Setting 3**

An initial set of 4000 simulations was filtered to extract those where after 10 years of MDA, the prevalence of TF in children aged 1-9 years (TF_1-9_) was more than 10%, giving a subset for analysis of at least 1000 simulations for each considered value of the adherence-correlation parameter $\rho$ (higher values of $\rho$ were not explored as they were considered unrealistic). Distributions of TF_1-9_ at baseline, and in 2030 are given in Figure S1, considering three possible strategies post-2020 (assuming a 2020 interruption to MDA). These are continuing annual MDA as previously, (scenario II in main text), and mitigation and acceleration strategies MA1 and MA2 as described in main text.

**Table S3. Summary of Setting 3 at baseline. 95% Confidence intervals (CI) are given as 95^th^ centiles.**

|  | **Adherence-correlation parameter** $\boldsymbol{\rho}$ | **Median prevalence**  **(95% CI)** | **Mean**  **prevalence (s.d)** |
| --- | --- | --- | --- |
| **Baseline** | 0 | 43.5 (31.0,54.5) | 43.2 (6.1) |
|  | 0.3 | 43.3 (30.6,54.6) | 43.1 (6.32) |
|  | 0.5 | 42.0 (30.1, 54.7) | 42.9 (6.34) |

It is acknowledged that the baseline prevalence estimates for Setting 3 are likely to be lower than those observed in settings where control has not been achieved after more than 10 years of MDA. This is likely due to the fact that the initial model was fitted and parameterised using data from settings where rates of transmission is such that annual MDA has largely been successful.^2,3,12^ The implications of this are that some model assumptions (for example reduced infection/disease duration being a function of infection history and acquired immunity alone and not a combination of age and infection history) and parameter estimations are less representative of very high transmission settings. This means that in such settings, the non-linearity in the model of the relationship between the transmission parameter and baseline TF may mean baseline TF is underestimated. However the qualitative trends (equilibrium despite annual MDA, shift in this equilibrium with enhanced MDA) are still relevant. Further model fitting to relevant data is anticipated to improve the realism of the assumptions and parameter estimates for very high transmission settings.

**
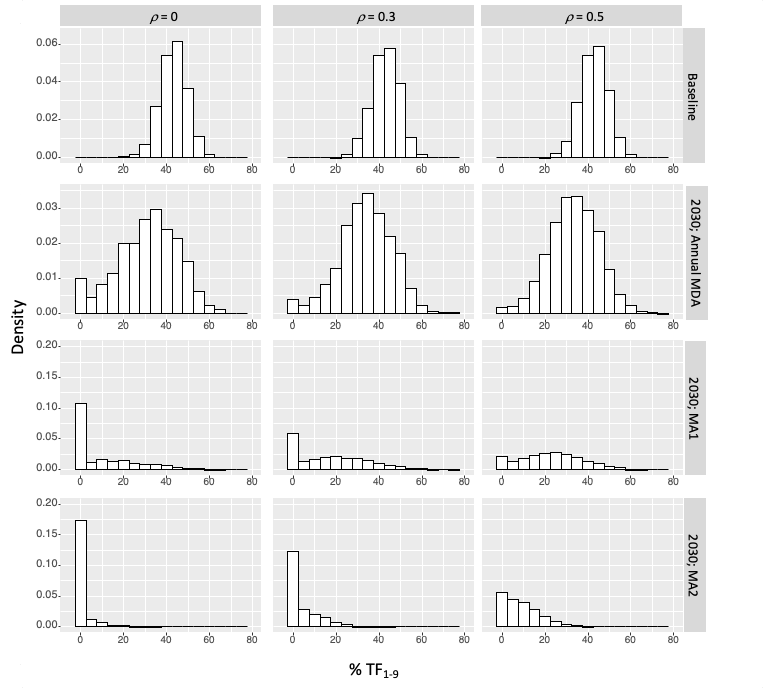
**

**Figure S1. Distributions of TF prevalence in children aged 1-9 years (% TF_1-9_) at baseline (row 1) and in 2030 (rows 2-4).** 2030 distributions follow simulation of either annual MDA beyond the 2020 interruption (row 2), or mitigation and acceleration strategies MA1 (row 3) or MA2 (row 4).

**Additional results: 2 year interruption to MDA.**

**Table S4: Summary of model output for Setting 1 and Setting 2.** 48 month interruption to MDA. Confidence intervals are given as 95th centiles.

| **Setting 1** | **Mean years to achieve EPHP (Median; 95% CI)** | **% simulations reaching TF_1-9_<5% after 6 rounds of MDA** | **Mean % TF in children after 6 rounds MDA (Median; 95% CI)** |
| --- | --- | --- | --- |
| **I) No interruption** | 4.3 (4.1; 2.4-11.1) | 86.5 | 1.8 (0; 0-12.3) |
| **II) 2020 & 2021**  **interruption;**  **No mitigation** | 9.4 (8.2; 2.4->16^a^) | 55.7 | 5.9 (3.8; 0-22.3) |
| **M1) 2020 & 2021**  **interruption;**  **Extra MDA round in**  **2022: Community** | 6.5 (6.5; 2.4->16^a^) | 75.1 | 3.2 (0.6; 0-17.3) |
| **M1) 2020 & 2021**  **interruption;**  **Extra MDA round in**  **2022: Children** | 8.4 (7.1; 2.4->16^a^) | 56.7 | 5.7(3.8; 0-21.6) |
| **Setting 2** | **Mean years to achieve EPHP (Median; 95% CI)** | **% simulations reaching TF_1-9_<5% after 4 rounds of MDA** | **Mean % TF in children after 4 rounds MDA (Median; 95% CI)** |
| **I) No interruption** | 2.7(2.6; 1.7-4.6) | 98.6 | 0.8(0.4; 0-4.2) |
| **II) 2020 & 2021**  **interruption;**  **No mitigation** | 5.2(5.1; 1.7-7.6) | 95.1 | 1.4(0.8; 0-6.5) |
| **M1) 2020 & 2021**  **interruption;**  **Extra MDA round in**  **2022: Community** | 5.0(4.9; 1.7-5.8) | 98.9 | 1.0(0.4; 0-4.2) |
| **M1) 2020 & 2021**  **interruption;**  **Extra MDA round in**  **2022: Children** | 5.1(5.0; 1.7-6.4) | 94.0 | 1.8(1.2; 0-7.2) |

^a^TF_1-9_ is not reached within the timescale of the simulations (16 years).

**Table S5. Policy-Relevant Items for Reporting Models in Epidemiology of Neglected Tropical Diseases (PRIME-NTD).**^15^

| **Policy Relevant Principle** | **Application to manuscript** | **Location of specific detail** |
| --- | --- | --- |
| Stakeholder engagement | Work has been presented at the following WHO webinars: (i) Neglected Tropical Diseases and COVID-19: Impact on Programme Implementation; and (ii) A Research Agenda for NTD Programmes Affected by the COVID-19 Pandemic. Dialogue with stakeholders including BMGF, TCC and ITI has refined work to ensure policy relevance. |  |
| Complete model documentation | Transmission model described and code is available in GitHub repository | Methods/Supplementary data  Github: <https://github.com/AnnaMB123/AnnaMB123_TRSTMH_Trachoma> |
| Complete description of data used | Simulations not fitted to data; parameters are taken from the literature, sources specified in the supplementary material | Methods/Supplementary data |
| Communicating uncertainty | Assumptions of the model are described and their implications/limitations are discussed. | Methods/Discussion |
| Testable model outcomes | No data currently available to validate the model, however results are similar to a similar model presented in this special collection. | ^16^ |

WHO= World Health Organisation

BMGF= Bill and Melinda Gates Foundation

TCC= The Carter Center

ITI=International Trachoma Initiative

**Glossary of terms**

| **Term** | **Definition** |
| --- | --- |
| MDA | Mass drug administration |
| Azithromycin | Antibiotic used in MDA for treatment of trachoma |
| EPHP | Elimination as a public health problem |
| TF | Trachomatous inflammation—follicular  (a clinical sign of active trachoma diagnosed via clinical examination with a magnifying loupe). |
| TF_1-9_ | Prevalence of TF in children aged 1-9 years. |
| *Chlamydia trachomatis* | The bacterial causative agent of trachoma infection |
| *R_0_* | The basic reproductive number (mean number of secondary infections generated by the average infected person in a susceptible population) |
| *R_T_* | The basic reproductive number under treatment (mean number of secondary infections generated by the average infected person in a population that is receiving annual community-wide MDA with azithromycin) |
| Hypoendemic | TF prevalence in ages 1-9 years 5-9.9% |
| Mesoendemic | TF prevalence in ages 1-9 years 10-29.9% |
| Hyperendemic | TF prevalence in ages 1-9 years ≥30% |

**Supplementary Data: References**

1. Pinsent A, Hollingsworth TD. Optimising sampling regimes and data collection to inform surveillance for trachoma control. PLoS Negl Trop Dis. 2018;12(10):e0006531. doi:10.1371/journal.pntd.0006531

2. Pinsent A, Gambhir M. Improving our forecasts for trachoma elimination: What else do we need to know? PLoS Negl Trop Dis. 2017;11(2):e0005378. doi:10.1371/journal.pntd.0005378

3. Bailey R, Duong T, Carpenter R, Whittle H, Mabey D. The duration of human ocular Chlamydia trachomatis infection is age dependent. Epidemiol Infect. 1999;123(3):479-486. doi:10.1017/s0950268899003076

4. Bailey RL, Arullendran P, Whittle HC, Mabey DC. Randomised controlled trial of single-dose azithromycin in treatment of trachoma. Lancet (London, England). 1993;342(8869):453-456. doi:10.1016/0140-6736(93)91591-9

5. Lakew T, House J, Hong KC, et al. Reduction and return of infectious trachoma in severely affected communities in Ethiopia. PLoS Negl Trop Dis. 2009;3(2):e376. doi:10.1371/journal.pntd.0000376

6. Oldenburg CE, Amza A, Kadri B, et al. Comparison of Mass Azithromycin Coverage Targets of Children in Niger: A Cluster-Randomized Trachoma Trial. Am J Trop Med Hyg. 2018;98(2):389-395. doi:10.4269/ajtmh.17-0501

7. Godwin W, Prada JM, Emerson P, et al. Trachoma Prevalence After Discontinuation of Mass Azithromycin Distribution. J Infect Dis. 2020;221(Supplement_5):S519-S524. doi:10.1093/infdis/jiz691

8. World Health Organization. Report of the 3rd Global Scientific Meeting on Trachoma; 2010. Balitmore USA. https://www.who.int/blindness/publications/WORLDHEALTHORGANIZATIONGSMmtgreportFINALVERSION.pdf?ua=1 (accessed 30^th^ October 2020)

9. Solomon AW, Holland MJ, Burton MJ, et al. Strategies for control of trachoma: observational study with quantitative PCR. Lancet (London, England). 2003;362(9379):198-204. doi:10.1016/S0140-6736(03)13909-8

10. Lietman TM, Deiner MS, Oldenburg CE, Nash SD, Keenan JD, Porco TC. Identifying a sufficient core group for trachoma transmission. PLoS Negl Trop Dis. 2018;12(10):e0006478. doi:10.1371/journal.pntd.0006478

11. Dyson L, Stolk WA, Farrell SH, Hollingsworth TD. Measuring and modelling the effects of systematic non-adherence to mass drug administration. Epidemics. 2017;18:56-66. doi:10.1016/j.epidem.2017.02.002

12. Grassly NC, Ward ME, Ferris S, Mabey DC, Bailey RL. The natural history of trachoma infection and disease in a Gambian cohort with frequent follow-up. PLoS Negl Trop Dis. 2008;2(12):e341. doi:10.1371/journal.pntd.0000341

13. Shattock AJ, Gambhir M, Taylor HR, Cowling CS, Kaldor JM, Wilson DP. Control of trachoma in Australia: a model based evaluation of current interventions. PLoS Negl Trop Dis. 2015;9(4):e0003474. doi:10.1371/journal.pntd.0003474

14. Liu F, Porco TC, Mkocha HA, et al. The efficacy of oral azithromycin in clearing ocular chlamydia: mathematical modeling from a community-randomized trachoma trial. Epidemics. 2014;6:10-17. doi:10.1016/j.epidem.2013.12.001

15. Behrend MR, Basáñez M-G, Hamley JID, et al. Modelling for policy: The five principles of the Neglected Tropical Diseases Modelling Consortium. PLoS Negl Trop Dis. 2020;14(4):e0008033. doi:10.1371/journal.pntd.0008033

16. Blumberg S, Borlase A, Prada JM, Solomon AW, Emerson P, Hooper PJ, Deiner MS, Amoah B, Hollingsworth TD, Porco TC, Lietman TM. Implications of the COVID-19 pandemic on eliminating trachoma as a public health problem. Under Review. Published online 2020.
